# Morphologic characterization and cytokine response of chicken bone-marrow derived dendritic cells to infection with high and low pathogenic avian influenza virus

**DOI:** 10.3389/fimmu.2024.1374838

**Published:** 2024-08-30

**Authors:** Jongsuk Mo, Karen Segovia, Klaudia Chrzastek, Kelsey Briggs, Darrell R. Kapczynski

**Affiliations:** ^1^ Exotic and Emerging Avian Disease Research Unit, U.S National Poultry Research Center, Agricultural Research Service, United States Department of Agriculture (USDA), Athens, GA, United States; ^2^ CSL Seqirus, Waltham, MA, United States; ^3^ Pathology and Animal Sciences, Animal and Plant Health Agency (APHA), Addlestone, United Kingdom

**Keywords:** chicken, innate immunity, dendritic cells, avian influenza, cytokines, interferon

## Abstract

Dendritic cells (DCs) are professional antigen-presenting cells, which are key components of the immune system and involved in early immune responses. DCs are specialized in capturing, processing, and presenting antigens to facilitate immune interactions. Chickens infected with avian influenza virus (AIV) demonstrate a wide range of clinical symptoms, based on pathogenicity of the virus. Low pathogenic avian influenza (LPAI) viruses typically induce mild clinical signs, whereas high pathogenic avian influenza (HPAI) induce more severe disease, which can lead to death. For this study, chicken bone marrow-derived DC (ckBM-DC)s were produced and infected with high and low pathogenic avian influenza viruses of H5N2 or H7N3 subtypes to characterize innate immune responses, study effect on cell morphologies, and evaluate virus replication. A strong proinflammatory response was observed at 8 hours post infection, via upregulation of chicken interleukin-1β and stimulation of the interferon response pathway. Microscopically, the DCs underwent morphological changes from classic elongated dendrites to a more general rounded shape that eventually led to cell death with the presence of scattered cellular debris. Differences in onset of morphologic changes were observed between H5 and H7 subtypes. Increases in viral titers demonstrated that both HPAI and LPAI are capable of infecting and replicating in DCs. The increase in activation of infected DCs may be indicative of a dysregulated immune response typically seen with HPAI infections.

## 1 Introduction

In recent years, avian influenza virus (AIV) has been one the leading causes of infection-based poultry mortality and morbidity. Prior to the 1990s, AIV outbreaks in domesticated poultry were rare, however ongoing outbreaks of highly pathogenic avian influenza (HPAI) have occurred globally for the past several years (1–3). The H5 A/goose/Guangdong/1996 (H5-Gs/Gd) lineage is responsible for most of the outbreaks, as the current clade 2.3.4.4b viruses appear to be highly adapted to migratory waterfowl (3). As a result of the adaptation, more spill over into domesticated poultry, mammals, and humans have been observed (4). High morbidity and mortality rates have led to reduced poultry production, embargoes on countries of origin, and increased expenses associated with vaccinating and controlling AIV within the global poultry industry (5, 6). In 2022, a total of 67 countries reported HPAI outbreaks, resulting in the deaths of 131 million poultry and wild birds (7, 8). In the U.S., the ongoing 2022-2024 HPAI H5N1 outbreak has resulted in the loss of over 60 million birds and $3 billion dollars in economic damages (1, 9).

Low pathogenic avian influenza (LPAI) viruses typically cause a mild disease in poultry that is restricted to the respiratory and intestinal tract because they contain a mono-basic cleavage site in the hemagglutinin (HA) protein that can only be cleaved by a few, localized cellular proteases (6, 10, 11). HPAI viruses contain a multi-basic cleavage site that allows for several common proteases to cleave the HA, which leads to a severe, systemic infection (6, 11). The rapid, multi-organ infection coupled with HPAI-specific dysregulated cytokine responses typically lead to death 1-6 days post infection in domesticated poultry (12). Early responses against viral infections are pre-dominantly mediated by host innate immunity, followed by migration of antigen-presenting cells (APC) and lymphocytes into the lymphoid tissues to initiate adaptive immune responses. Increased expression of pathogen recognition receptors (PRRs), interferons, pro-inflammatory cytokines, and chemokines are generally observed during the early stages of an AIV infection (13). PRRs, such as Toll-like receptors (TLRs) and MDA-5, sense viral RNAs and initiate inflammatory responses by releasing proinflammatory cytokines (14). A rapid induction of type 1 (interferon-alpha (IFN-α)) and type 2 (interferon-beta (IFN-β)) interferon leads to the upregulation of interferon stimulated genes (ISG)s, which are essential for an antiviral response. In particular, myxovirus resistance gene (Mx) is important because it promotes anti-AIV activity in various mammalian and avian species (14–17). The role of Mx is contested in chickens as there are conflicting reports of its effectiveness against HPAIV, however there is a known interaction between the viral nucleoprotein (NP) and Mx proteins (14, 17–20). Proinflammatory cytokines, including interleukin 6 (IL-6), interleukin 12 (IL-12), and interleukin 1 beta (IL-1β) upregulate inflammatory cytokine responses to limit infection, while anti-inflammatory cytokines such as IL-10 can inhibit expression of proinflammatory cytokines to down regulate the inflammation process (21).

Several AIV proteins have been implicated activating the necrotic and apoptotic cell death pathways (22). Necrosis is a passive, uncontrolled cell death, which typically causes an inflammatory reaction and affects surrounding cells, whereas apoptosis is an active, controlled cell death that does not affect surrounding cells (12, 22, 23). While both can occur during an infection, AIV proteins have been shown to block the Caspase-3 (Casp-3) and Caspase-8 (Casp-8) activation causing a shift from the apoptotic pathway to the necrotic pathway (24–30). The expression of these innate immune modulators drastically varies by virus strain, host, and target tissue making our understanding of immune response to AIV incomplete (12, 13).

APCs are crucial components of the primary immune response against pathogens and help bridge the innate and adaptive immune responses. Dendritic cells (DC) are professional APCs that play a central role as regulators of the adaptive immune response by interacting with T and B cells (13). Avian DC progenitors originate from hematopoietic stem cells in the bone marrow and translocate to non-lymphoid tissue where they become immature DCs (13, 31, 32). While immature DCs are capable of phagocytizing antigens, they are poor T-cell stimulators and lack proper antigen presentation capabilities. Upon activation, chicken DCs migrate to T-cell regions where they mature and upregulate several costimulatory molecules, including MHC-II, CD11c, CD40 and CD80. Mature DCs are specialized in antigen presentation to T cells (33). Recently, more emphasis has been put on understanding the immune modulation of chicken DC cells and their ability to combat disease.

Previous studies reported DCs could be grown *in vitro* by incubating chicken bone marrow (BM)-derived cells with chicken granulocyte-macrophage stimulating factor (GM-CSF) and chicken interleukin 4 (IL-4) (34, 35). In this study, we cultured bone-derived chicken dendritic cells (ck-BM-DC) and examined gene expression levels of IFN-α, TLR-3, TLR-7, MHC-I, IL-1β, IL-6, Mx, Casp-3, and Casp-8 pre/post infection with AIV. There are limited studies examining the interactions between chicken DCs and AIV (32, 36–38). The exact nature of how AIV infections affect DCs is largely unknown. We seek to determine whether active AIV replication can occur in DCs and if antigen processing occurs. In this study, we compared immune responses, morphological changes, and replication of ckBM-DCs following infection with contemporary H5 and H7 HPAI and LPAI viruses. A better understanding of how chicken antigen presentation occurs is needed, as the Gs/Gd lineage becomes entrenched in migratory waterfowl globally.

## 2 Methods

### 2.1 Chickens and chicken bone marrow dendritic cells isolation and culture

Four-week-old specific pathogen-free (SPF) white leghorn chickens were housed at the USDA-ARS U.S. National Poultry Research Center. The studies involving animals were reviewed and approved by the USDA-ARS U.S. National Poultry Research Center Institutional Animal Care and Use Committee (IACUC). All birds used in these studies were cared for and handled in compliance with IACUC guidelines and procedures. ckBM-DCs were generated as previously described with minor modifications to the protocol (35). Briefly, following euthanasia with injected sodium pentobarbital using AVMA guidelines, femurs of chickens were removed and placed into 10 cm petri dishes containing 1X PBS with 1% antibiotics (Sigma-Aldrich, St. Louis, MO). Both ends of the femur bone were cut across the tops with sterile bone-scissors and a sterile iron wire was passed through and the bone marrow was flushed with sterile 1X PBS using 20 ml syringe with 16G needle. Marrow clusters were gently meshed through a 70 nm screen using a syringe plunger to obtain single-cell suspensions. Cell suspensions were overlaid with an equal volume of Histopaque 1119 (Sigma-Aldrich, St. Louis, MO) and centrifuged at 1200 g for 30 min at RT to remove red blood cells. Cells were collected and were washed three times in RPMI-1640 media (Thermo-fisher Scientific, Waltham, MA). After collection, cells were resuspended in 1X PBS and mixed 1:1 with trypan blue solution (Thermo-fisher Scientific, Waltham, MA) and checked under microscope using a hemacytometer for viable cells.

Cells were cultured in six-well plates at a concentration of 2×10^6^ cells/ml at 41°C 5% CO_2_ in RPMI-1640 supplemented with 10% chicken serum (Thermo-fisher, Waltham, MA), 1% L-glutamine, 1% non-essential amino acids and antibiotics (Gibco, Thermo-fisher, Waltham, MA) for 7 days. Different concentrations (0, 10, 25 and 50 ng/ml) of yeast-produced recombinant chicken IL-4 and chicken GM-CSF (Kingfisher, St Paul, MIM) were added to the medium to optimize culture conditions. Fresh complete medium was mixed with conditional media at a 3:1 ratio and added to the cells every 2 days. To induce maturation of bone-marrow cells into DCs, cells were stimulated with *Escherichia coli* LPS (500 ng/ml) (Thermo-fisher Scientific, Waltham, MA) for 30 hours. Images of the cells were taken at 30 hours using an EVOS 5000 (Invitrogen, Carlsbad, CA).

### 2.2 Virus propagation and inactivation

A total of 7 viruses, HPAI H5N2 A/chicken/Pennsylvania/1370/1983, H7N3 A/chicken/Jalisco/CPA1/2012 and LPAI H5N2 A/chicken/Pennsylvania/21525/1983, A/Cinnamon Teal/Mexico/2817/2006 (H7N3), A/turkey/Virginia/SEP-4/2009 (H1N1) and A/Turkey/Wisconsin/68 (H5N9) strains were propagated in the allantoic cavities of 9–11-day old SPF chicken eggs. Viral titers were determined as previously described (39). The H5N9 strain was inactivated with 0.01% β-propiolactone (BPL) overnight, followed by dialysis with sterile 1X PBS. Inactivation of the virus was tested by performing serial passages on eggs. After inactivation, the strain was labeled with FITC labeling kit according to manufacturer`s recommendation (Thermo-fisher, Waltham, MA.)

### 2.3 Morphology and phenotypic analysis

Cells were cultured for 6 days in the presence of different concentrations (0, 10, 25 and 50 ng/ml) of chicken GM-CSF and chicken IL-4. Cell morphology and cell growth were monitored daily. After stimulation with *Escherichia coli* LPS (500ng/ml) for 30 hours, images were taken to check change in cell morphology. Immunofluorescence labelling was performed to analyze the DC markers using FITC labeled mouse-anti-chicken MHC-II in combination with mouse anti-chicken CD40 (Bio-Rad, Hercules, CA), mouse anti-chicken CD11c (8F2, IgG2a) followed by incubation with a goat anti-mouse-Ig secondary Ab (Thermo-fisher Scientific, Waltham, MA). Primers for surface markers (MHC-II, CD40, CD11c, CD80, CD83 and CD86) were designed according to methods we have previously used (28).

### 2.4 Phagocytosis assay

Phagocytosis was assessed using FITC labeled inactivated H5N9 virus, and 0.5µm carboxylate modified fluorescent red latex beads (Sigma-Aldrich, St. Louis, MO). To explain briefly, non-stimulated ckBM-DCs were cultured for 6 days, followed by incubation with FITC-labeled inactivated H5N9 virus or chicken serum-opsonized red latex beads in RPMI-1640 medium at a density of 10^8^ particles/ml at 41°C for 4 hours. Cells were washed five times with 1X PBS and visualized with immunofluorescence microscopy.

### 2.5 Immunofluorescence analysis

For sialic acid receptor staining, cells were fixed and stained by incubating FITC-labeled MAA (SA-α2,3-Gal) and TRITC-labeled SNA (SA-α2,6-Gal) for 1 hour at room temperature. Following 3 rinses in 1X PBS, cells were stained for 5 minutes with DAPI (Thermo-fisher Scientific, Waltham, MA). The immunofluorescence assays for virus nuclear protein (NP) detection were performed as previously described (40). Briefly, cells were infected with A/turkey/Virginia/SEP-4/2009 (H1N1) and A/Turkey/Wisconsin/68 (H5N9) virus at a MOI of 1 for 20 hours. Cells were then washed with 1X PBS twice, fixed and permeabilized with methanol. Viral antigens were detected with mouse-derived monoclonal antibody specific for a type A influenza virus nucleoprotein (developed at Southeast Poultry Research Laboratory, USDA), then stained with FITC-conjugated anti-mouse IgG antibody (Thermo-fisher Scientific, Waltham, MA).

### 2.6 Virus infection and analysis of cytokine expression by quantitative real-time RT-PCR

Cells were infected with either LPAI or HPAI H5N2 and H7N3 at a MOI of 1 in serum free DC medium for one hour with gentle agitation applied every 10 minutes. Cells were washed twice with 1X PBS and resuspended in DC medium containing 2% chick serum and incubated at 41°C 5% CO2. At 2, 8 and 24 hours post infection (hpi), supernatants were collected and stored at -80°C until titration. Virus titers are expressed as log10 50% embryo infectious dose (EID_50_/ml) and HAU. Cells were harvested for RNA extraction at 8 hpi. Relative gene expression levels of IFN-α, Mx, TLR-3, TLR-7, MHC-I, IL-1β, IL-6, Casp-3 and Casp-8 were evaluated by qRT-PCR as previously described (28).

### 2.7 Statistical analyses

Data are expressed as the mean ± standard error. Statistical differences were analyzed with Tukey one-way ANOVA using Prism 9 (GraphPad Co., San Diego, CA).

## 3 Results

### 3.1 Morphological characteristics of chicken bone marrow-derived DC

**Figure 1:**
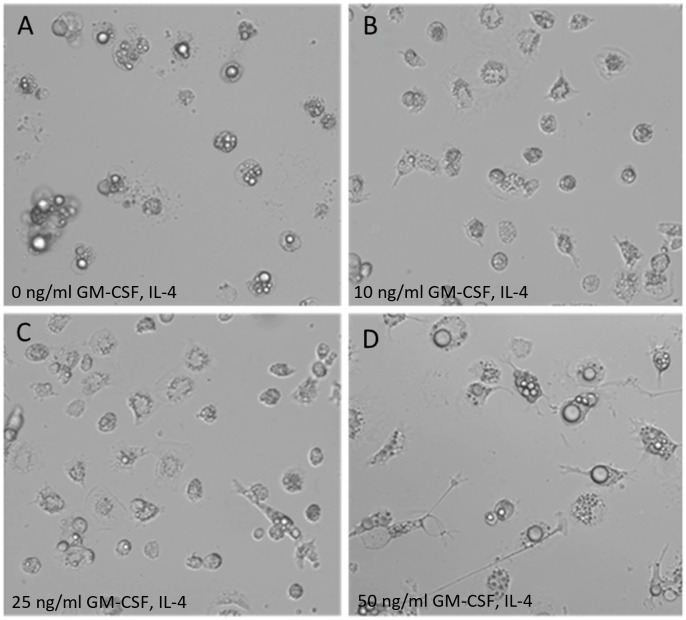
Morphology of ckBM-DC. Bone marrow-derived cells were cultured in the presence of different levels of recombinant chicken granulocyte-macrophage stimulating factor (GM-CSF) and recombinant chicken interleukin-4 (IL-4) for 6 days and dendrite formation was observed by microscopy. **(A)** 0 ng/ml GM-CSF + 0 ng/ml IL-4. **(B)** 10 ng/ml GM-CSF + 10 ng/ml IL-4. **(C)** 25 ng/ml GM-CSF + 25 ng/ml IL-4. **(D)** 50 ng/ml GM-CSF + 50 ng/ml IL-4. A representative image is shown for each concentration at 200x magnification.

Morphological characteristics of ckBM-DC differed based on the levels of recombinant chicken GM-CSF and IL-4 (0, 10, 25, and 50 ng/ml) used. Untreated bone marrow cells displayed a rounded appearance, with follicle-like structures (cytoplasmic vacuoles) present within the cytoplasm (**Figure 1A**). Cells treated with 10 ng/ml or 25 ng/ml of GM-CSF and IL-4 retained a rounded appearance, but a few cells were observed to have some elongated morphology (**Figures 1B, C**). Cells treated with 50 ng/ml exhibited the greatest DC-like morphology by morphing into larger, elongated, and branched cells, as previously described (**Figure 1D**) (35). While no international consensus exists on how to determine units of activity for avian cytokines, 50 ng/ml of GM-CSF and 50 ng/ml IL-4 were used to maximize the number of cell aggregates in this study.

### 3.2 Maturation of ckBM-DC

**Figure 2:**
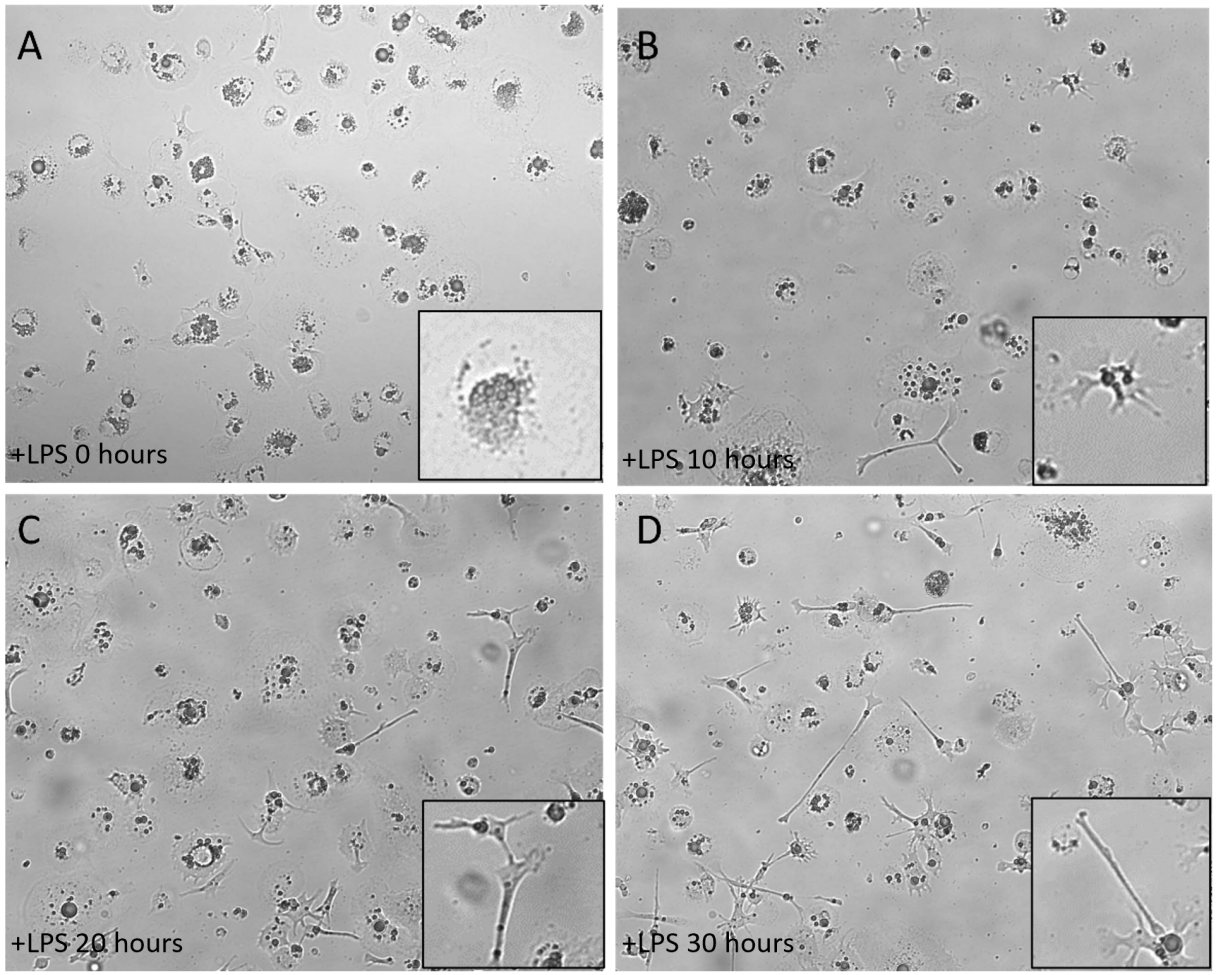
Morphology of immature ckBM-DC stimulated with LPS. Cells were cultured in the presence of 50 ng/ml GM-CSF + 50 ng/ml IL-4 for 6 days and then stimulated with LPS (500ng/ml). ckBM-DCs were observed by microscopy for 30 hours. Images show cells cultured at **(A)** 0 hours, **(B)** 10 hours, **(C)** 20 hours, and **(D)** 30 hours. A representative image is shown for each timepoint at 100x magnification. Differening levels of elongated dendrites are in black boxes.

To induce maturation of the ckBM-DCs, we stimulated cells with 500 ng/ml LPS on day 6 post culture for 30 hours. The cells were examined at different timepoints (0, 10, 20, and 30 hours) after the addition of LPS. At the 0 timepoint, cells displayed a veiled appearance, with small elongated branches on each cell (**Figure 2A**). After incubating with LPS for 10 hours, the DCs began displaying long and thin branch-like features, with a spiny or sheet-like appearance (**Figure 2B**). At the later timepoints, 20 and 30 hours, most of the cells developed the dendritic-like appearance (dendrites), indicating the presense of activated, mature DCs (**Figure 2C, D**).

### 3.3 Mature ckBM-DC cells share phenotypic similarities with mammalian DC cells

**Figure 3:**
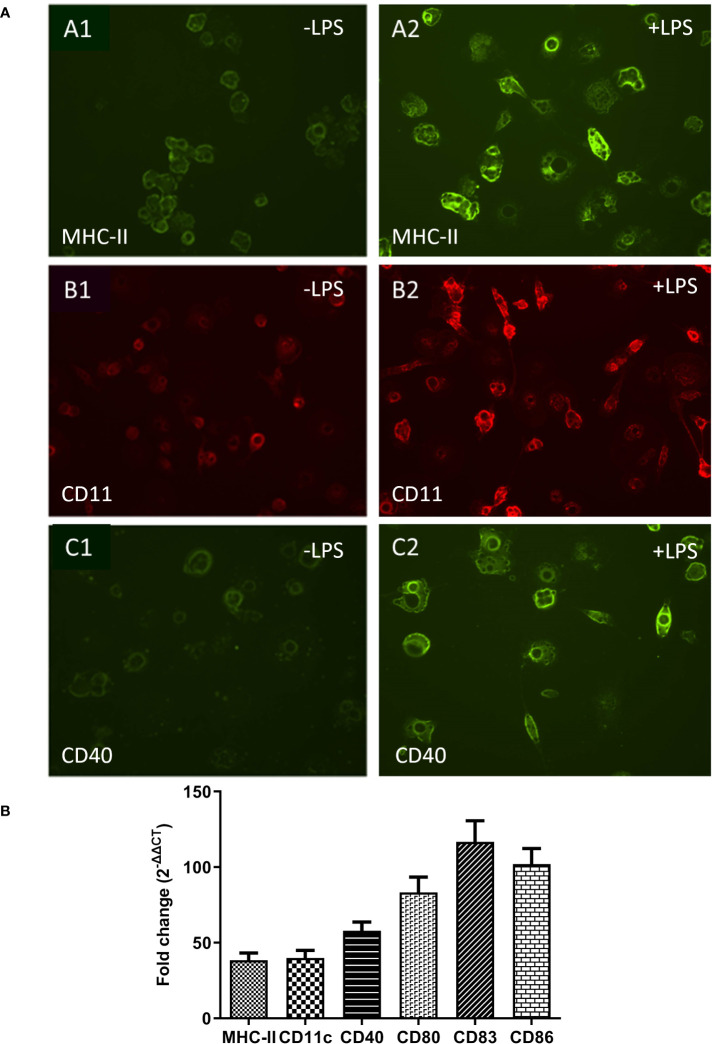
Comparative analysis of surface markers on immature and mature ckBM-DCs. Cells were cultured in the presence of 50 ng/ml GM-CSF + 50 ng/ml IL-4 for 6 days, and then stimulated with 500 ng/ml LPS for 30 hours. **(A)** Immature cells (-LPS) are on the left **(A1, B1, C1)** and mature cells (+LPS) are on the right **(A2, B2, C2)**. Immunofluorescence analysis was performed using a FITC labeled mouse-anti-chicken MHC-II antibody (green) **(A1, A2)**. Cells were also stained with mouse anti-chicken CD11c (B1, B2) (red) and mouse anti-chicken CD40 **(C1, C2)** followed by a goat-anti-mouse secondary (green). A representative image is shown for each at 100x magnification. **(B)** Cellular RNA was extracted to measure expression levels of surface markers, via qPCR, in ckBM-DCs. RNA was normalized using the Ck 28S house-keeping gene. The data is expressed as the fold change in mRNA levels between immature and mature ckBM-DCs for MHC-II, CD11c, CD40, CD80, CD83, and CD86. The data shown is a representative of three independent experiments. Error bars represent the standard error.

Dendritic cells co-exist in both immature and mature states. In mammals, immature dendritic cells are characterized by moderate or low-level expression of surface markers molecules such as MHC-II, CD11c, CD40, CD80, CD83 and CD86 and increase upon maturation (41). Immunofluorescence microscopy demonstrated that immature ckBM-DCs had some level of surface marker expression when stained with anti-chicken MHC-II (**Figure 3A1**), anti-chicken CD11c (**Figure 3B1**) and anti-chicken CD40 (**Figure 3C1**). After stimulation with LPS for 24 hours, expression level was increased in all 3 markers, MHC-II (**Figure 3A2**), CD11c (**Figure 3B2**) and CD40 (**Figure 3C2**). To quantify the level of surface marker expression, qPCR was used to determine the fold change of expression between immature and mature DCs. The level of surface marker expression was significantly enhanced in mature ckBM-DC cells, approximately 40-120-fold compared with their immature counterparts. The greatest change was observed with CD80, CD83 and CD86 (**Figure 3B**).

### 3.4 Immature ckBM-DCs retain the capability to phagocytosize foreign antigens

**Figure 4:**
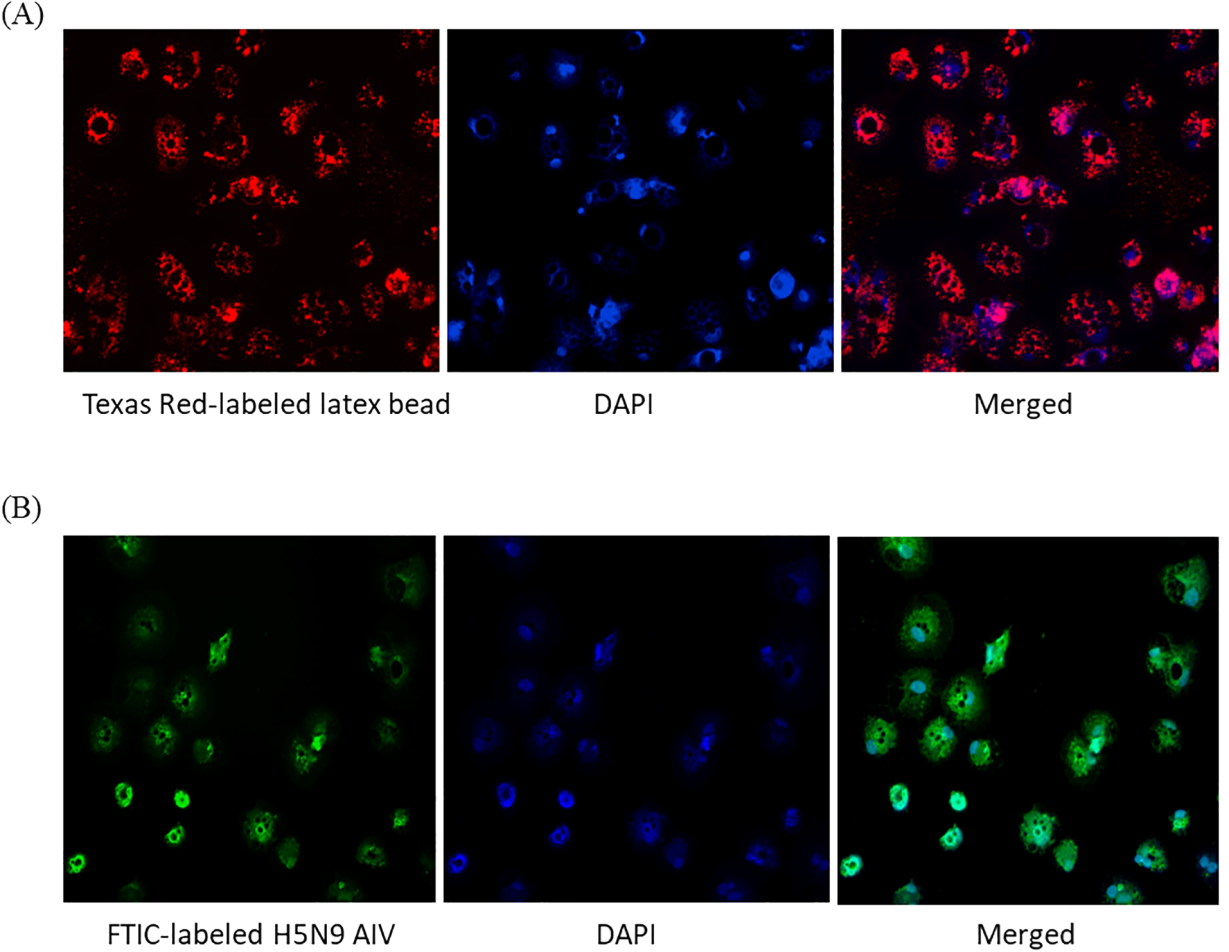
Functionality of immature ckBM-DCs. Cells were cultured in the presence of 50 ng/ml GM-CSF + 50 ng/ml IL-4 for 6 days. **(A)** ckBM-DCs were incubated with 0.5 µm carboxylate modified fluorescent red latex beads or **(B)** FITC labeled-inactivated H5N9 avian influenza virus for 4 hours. Following incubation cells were counterstained with DAPI, washed 5x with 1X PBS, and visualized by immunofluorescence microscopy. A representative image is shown for each at 100x magnification.

To test phagocytosis, 0.5 µm carboxylate modified fluorescent red latex beads and FITC labeled-inactivated H5N9 avian influenza virus particles were added to immature ckBM-DCs. The cells were able to phagocytose both the beads (**Figure 4A**) and viral particles (**Figure 4B**). The beads and virus were observed in the cytoplasm (**Figures 4A, B**).

### 3.5 AIVs are capable of infecting immature ckBM-DCs

**Figure 5:**
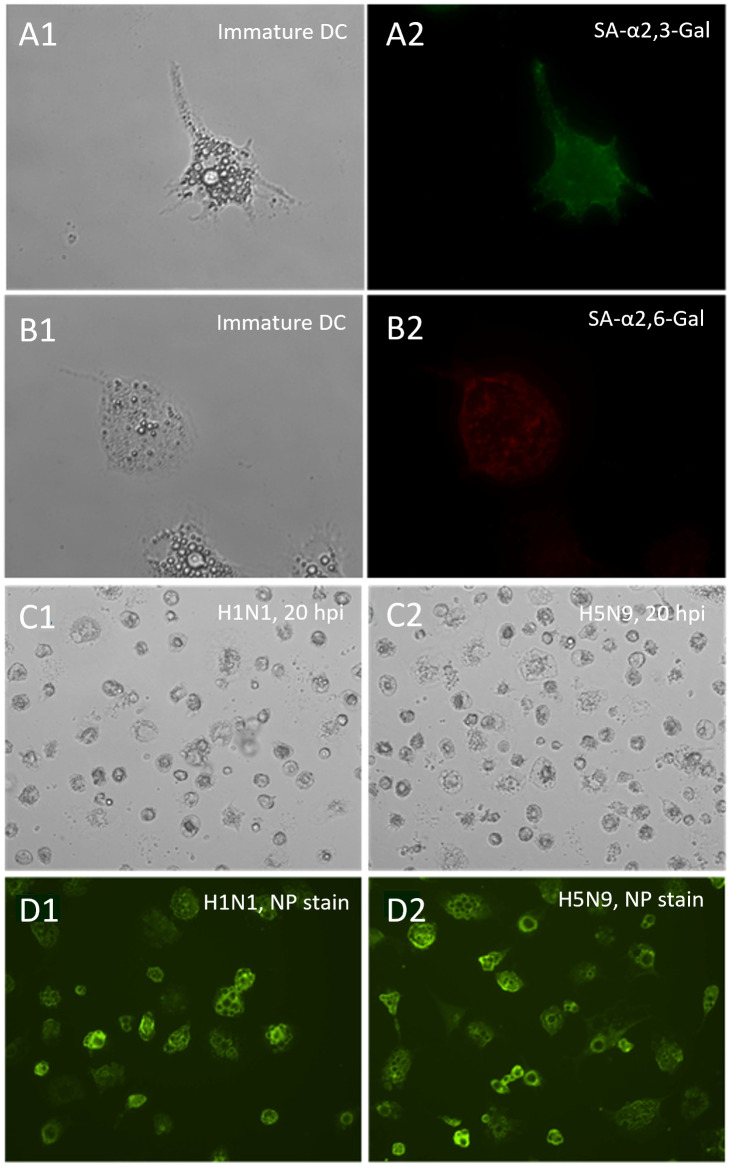
Distribution of sialic acid receptors on ckBM-DCs and susceptibility to pandemic H1N1 and H5N9 viruses. Cells were cultured in the presence of 50 ng/ml GM-CSF + 50 ng/ml IL-4 for 6 days. Immature ckBM-DCs **(A1, B1)** were stained with FITC-labeled MAA (SA-α2,3-Gal) **(A2)** or TRITC-labeled SNA (SA-α2,6-Gal) **(B2)** and visualized by immunofluorescence microscopy. CkBM-DCs were infected at an MOI of 1 with A/turkey/Virginia/SEP-4/2009 H1N1 (SA-α2,6-Gal preference) and A/turkey/Wisconsin/68 H5N9 (SA-α2,3-Gal preference). At 20 hpi, viral-infected cells, H1N1 **(C1)** and H5N9 **(C2)**, were washed 2x with 1X PBS, fixed with methanol, and observed by microscopy. Viral NP proteins, H1N1 **(D1)** and H5N9 **(D2)**, were detected using a mouse-anti-NP antibody followed by a FITC-conjugated anti-mouse IgG secondary **(D1,D2).** A representative image is shown for each at 200x **(A1,A2,B1,B2)** and 100x **(C1,C2,D1,D2)** magnification.

To test whether immature ckBM-DCs can successfully be infected with AIV, immunofluorescence microcopy was performed to detect expression of SA-α2,3-Gal and SA-α2,6-Gal receptors on the DCs surface. Results demonstrated that both SA-α2,3-Gal (**Figure 5A2**) and SA-α2,6-Gal (**Figure 5B2**) receptors were extensively expressed in the immature DCs, however it appears the SA-α2,3-Gal receptor was more prominent based on immunofluorescence (**Figure 5A2**). After infecting the cells with pandemic H1N1 (SA-α2,6-Gal preference) and H5N9 (SA-α2,3-Gal preference), immunofluorescence microscopy was performed using a AIV NP antibody to demonstrate the immature DCs could be infected with AIV (**Figure 5C, D**).

### 3.6 ckBM-DCs can be infected with both LPAIVs and HPAIVs

**Figure 6:**
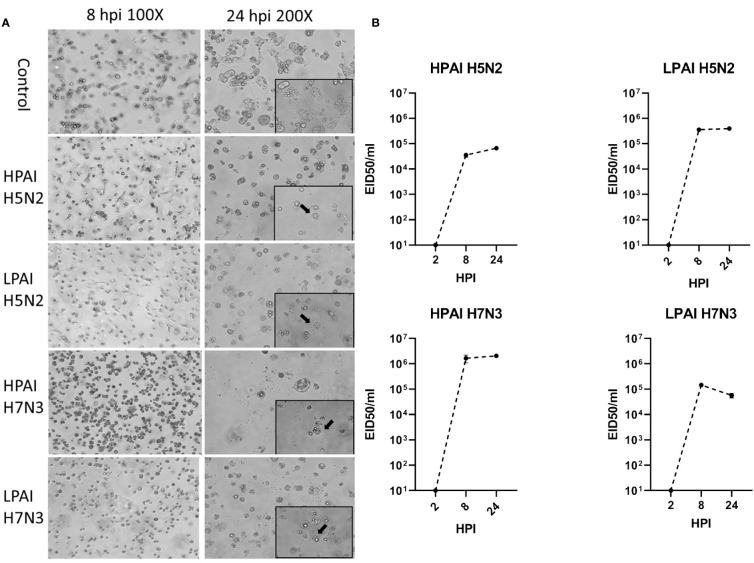
Change in morphology and growth of ckBM-DCs infected with LPAIV and HPAIV. Cells were cultured in the presence of 50 ng/ml GM-CSF + 50 ng/ml IL-4 for 6 days. Immature ckBM-DCs were infected at a MOI of 1 with LPAIV (A/Chicken/Pennsylvania/21525/1983 H5N2 and A/Cinnamon Teal/Mexico/2817/2006 H7N3) and HPAIV (A/Chicken/Pennsylvania/1370/1983 H5N2 and A/Chicken/Jalisco/CPA1/2017 H7N3) viruses. **(A)** CPE (black arrows) and cellular morphological changes were observed via microscopy at 8 and 24 HPI. A representative image is shown for each at 100x and 200x magnification. **(B)** Supernatants were obtained at 2, 8, and 24 HPI and viral titers were evaluated by EID50. The data shown is a representative of three independent experiments. Error bars represent the standard error of triplicate samples.

ckBM-DCs were infected with HPAIV and LPAIV (H5N2 and H7N3 subtypes) to determine the effect on cell morphology and viral replication. At 8 hpi, all infected cells underwent some degree of morphological change, more rounded cells were observed when infected with H7N3 compared to the H5N2 strains (**Figure 6A**). At 24 hpi, CPE was observed in the form of detached cells and changes in their morphology (rounding), regardless of subtype or pathogenicity (**Figure 6A**). There was little difference in severity of CPE between the H5N2 strains, but cells infected with HPAI H7N3 demonstrated more severe levels of CPE with larger number of detached cells compared to LPAI H7N3 (**Figure 6A**). In terms of viral growth, LPAI H5N2 demonstrated a titer of 10^5.5^ EID_50_/ml at 24 hpi, compared to HPAI H5N3 which demonstrated a titer of 10^4.8^ EID_50_/ml. In contrast, HPAI H7N3 demonstrated a higher titer compared to LPAI H7N3, with titers of 10^6.5^ EID_50_/ml and 10^4.8^ EID_50_/ml, respectively (**Figure 6B**). All virus titers increased by 3-5 logs between 2 and 8 HPI indicating AIV replicated in the ckBM-DCs (**Figure 6B**).

### 3.7 ckBM-DCs infected with HPAIVs demonstrates higher expression of immune markers compared to LPAIV

**Figure 7:**
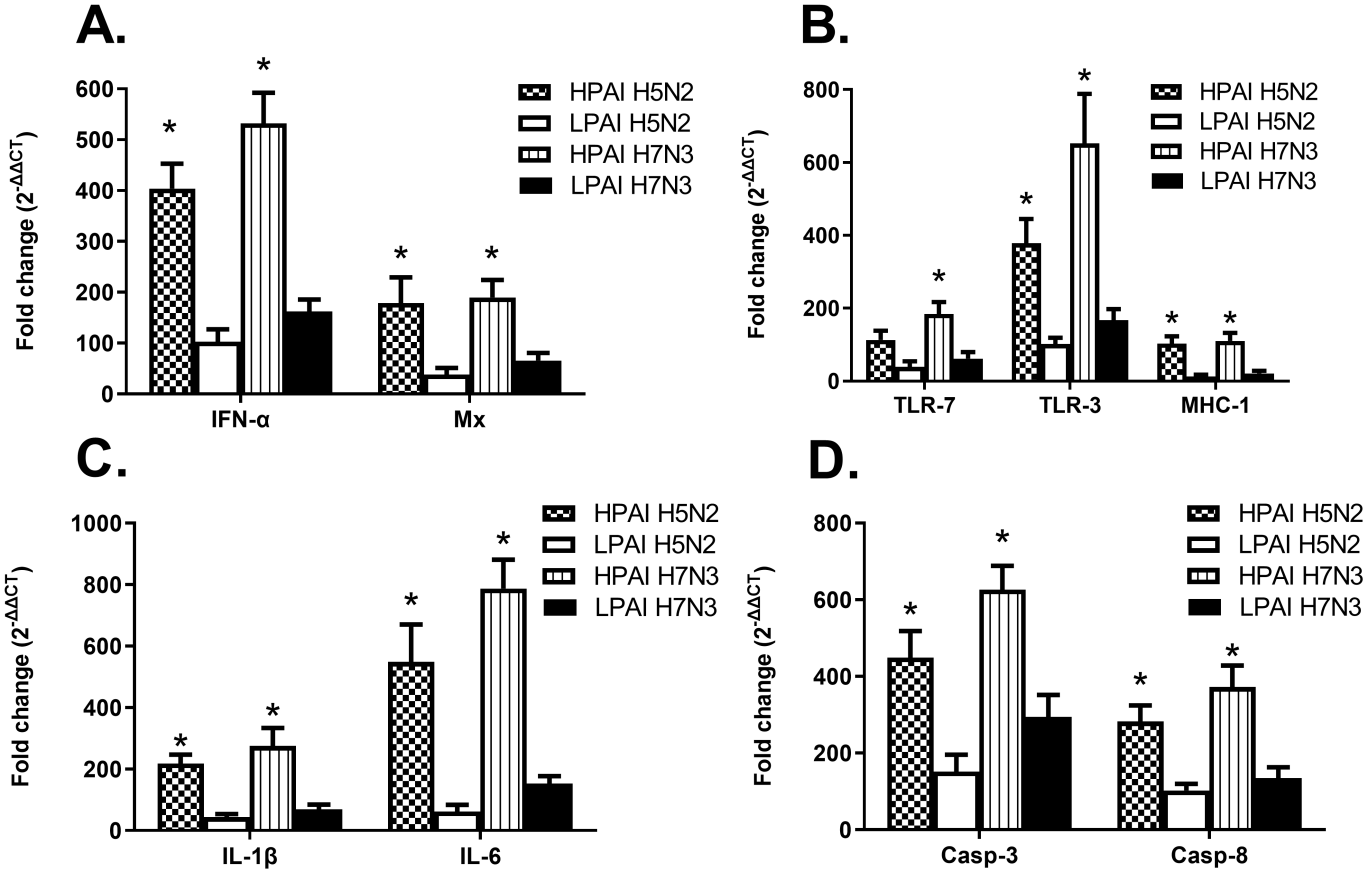
Cytokine expression levels of ckBM-DCs infected with HPAIV or LPAIV. Cells were cultured in the presence of 50 ng/ml GM-CSF + 50 ng/ml IL-4 for 6 days. Immature ckBM-DCs were infected at a MOI of 1 with LPAIV (A/Chicken/Pennsylvania/21525/1983 H5N2 and A/Cinnamon Teal/Mexico/2817/2006 H7N3) and HPAIV (A/Chicken/Pennsylvania/1370/1983 H5N2 and A/Chicken/Jalisco/CPA1/2017 H7N3) viruses. Cellular RNA was extracted 8 hpi to measure relative gene expression levels of IFNα, Mx, TLR-7, TLR-3, MHC-1, IL-1B, IL-6, Casp-3, and Casp-8 **(A–D)**. RNA was normalized using the Ck 28S house-keeping gene. The fold change (2^-ΔΔCT^) was determined by the comparison of infected ckBM-DCs to sham-infected ckBM-DCs. Tukey one-way ANOVA analysis was performed to determine significant differences between LPAIV and HPAIV infected ckBM-DCs. * indicates a significant difference (p<0.05). The data shown is a representative of three independent experiments. Error bars represent the standard error of triplicate samples.

Several immune markers were examined post AIV infection. Increased expression of IFN-α and Mx genes, which are indicative of a viral infection, were increased in all groups (**Figure 7**). All HPAI infected cells expressed significantly higher levels of both IFN-α and Mx genes, compared to their LPAI counterparts, between 20-500-fold, depending on virus and gene (**Figure 7A**). Increased expression of TLR receptors and MHC-I were observed in both HPAI and LPAI groups, with HPAI groups demonstrating higher expression levels compared to LPAI groups with a 10-700-fold difference. (**Figure 7B**). Similar trends were observed in gene expression levels of proinflammatory cytokines IL-1β, IL-6 and apoptotic genes Casp-3 and Casp-8, in which all HPAIV infected cells demonstrating significantly higher levels of expression compared to LPAI groups, between 20-800-fold difference. (**Figure 7C, D**).

## 4 Discussion

The innate immune system plays a central role in detecting viral pathogens and mounting an early response by activating inflammatory and antiviral defense mechanisms. DCs are essential in bridging the gap between innate and adaptive immune responses because they process and present antigens to T cells and B cells. However, it is still largely unknown if AIV can directly infect ckBM-DCs and if infection causes morphological and physiological changes to the cells. This study established that ck DCs can be infected by AIV, and that viral growth occurs in them. We also demonstrated ck-BMDCs were able to phagocytosize viral particles as immature DCs. The ckBM-DCs were able to be infected by both HPAIV and LPAIV isolates. However, differences in cell morphology did exist between the virus strains and pathogenicity. LPAI H5N2 replicated better in ckBM-DCs than its HPAI counterpart. While the exact reason is not clear, it may indicate a delayed replication in DCs or be attributed to HPAI H5N2 lack of adaptation in cell culture. In contrast, HPAI H7N3 demonstrated more severe CPE at 8 hpi compared to the LPAI H7N3, suggesting some correlation with pathogenicity and CPE with H7 viruses. Viral titers also correlated with CPE and pathogenicity, with the titer of HPAI H7N3 demonstrating a 1.7 log difference in EID_50_/ml titers compared to LPAI H7N3. The results are consistent with a previous study in which HPAI H7N1 showed better replication in chicken DCs compared to LPAI (32).

Controlled cell death is normally induced by apoptotic genes, during a viral infection up-regulation of related caspase genes is typically observed (23). In our study, Casp-3 and Casp-8 expression increasing in all infected groups, regardless of pathogenicity or subtype. However, DCs infected with HPAI viruses demonstrated significantly higher expression levels of Casp-3 and Casp-8 compared to their LPAI counterparts, indicating a correlation between caspase gene expression and pathogenicity. Studies have demonstrated AIV causes caspase-dependent apoptosis based on Casp-3 activation, which results in nuclear export of newly synthesized viral nucleoprotein (NP) and elevated virus replication. This suggests Casp-3 activation is a crucial event for AIV propagation and dissemination (42, 43). One study reported primary duck cells infected with LPAI H2N3 and classical H5N1 strains underwent rapid cell death compared to primary chicken cells, both cell lines showed similar levels of viral RNA, but lower amounts of infectious virus were observed in the duck cells (44). Such rapid cell death was not observed in the same study with duck cells infected with a contemporary Eurasian H5N1 strain fatal to ducks, indicating the rapid apoptosis may be part of a mechanism of host resistance against AIV (45). An increased expression of caspase genes demonstrated in our study may further support the notion that AIV can induce cell death via Casp-3 and Casp-8.

During AIV infection, ssRNA and dsRNA are recognized by a specific group of PRRs. In this study, HPAIV infected cells demonstrated significantly higher expression levels of TLR-3 and TLR-7 compared to cells infected with LPAIV. However, the level of TLR expression did not correspond to the amount of viral load as the titers had mixed results between the HPAI and LPAI strains. Furthermore, the TLR-3 expression levels were significantly higher in HPAI H7N3 compared to HPAI H5N2s. One study reported that TLR-3 expression levels significantly increased at 4 hpi and 16 hpi with HPAI H7N1 infections, whereas the level of increase in HPAI H5N2s were more gradual (32). TLR-3 and TLR-7 closely interact with STAT-3, which is crucial for regulating cytokine-mediated responses, such as IL-6 to combat viral infections (46). One study reported STAT-3 expression was not adversely affected by LPAIV H3N2 in chicken cells, but expression levels were significantly decreased in chicken cells infected by HPAI H5N1 (45). In contrast, STAT-3 expression levels were significantly elevated in duck cells, indicating infection with the same H5N1 strain had a less adverse effect in duck cells. Thus, it can be speculated that differences in the cell signaling process, along with specificity of the strains, may affect cytokine responses.

Our results demonstrated that expression levels of proinflammatory related genes were higher in the HPAI groups in the early stages of infection, compared to LPAI groups. Geus et al. reported that levels of IFN-α were elevated in HPAI infected DCs and were maintained up to 24 hpi, compared to the LPAI infected DCs where most of the IFN-α expression occurred in only the early stages (47). Our study demonstrated that the expression levels of IFN-α and IL-6 genes in DCs were higher in the HPAI H7N3 group, compared to the HPAI H5N2 group, suggesting the ability to activate host innate responses may vary depending on the virus subtype and the host. Several studies have reported high levels of IL-6, IL-12 and IL-18 cytokine expression in the lungs and spleens of chickens infected with H5 HPAIVs while type 1 interferons were mostly present in the plasma and tissues (48–51). Another study reported similar amounts of viral RNA and cytokine expression levels following infection with HPAI and LPAI H7N1 in chickens (52). Kuribayashi et al. (2013), demonstrated that H7N1 strains can replicate more efficiently in chickens compared to H7N7, especially in the brain and are able to trigger excessive expression of inflammatory and antiviral cytokines, such as IFN-γ, IL-1β, IL-6, and IFN-α, in proportion to its proliferation. In contrast, another study reported that human-origin DCs infected with HPAI H7 resulted in delayed and decreased expression of cytokines, including type 1 interferons, compared to other AIV subtypes (53). Thus, the difference of immune profiles of the host cell might be attributed to the specificity of the AIV. Furthermore, HPAI viruses may impair the regulatory activity of the TLR pathway, which is responsible for controlling the magnitude and duration of the inflammatory response, and lead to an uncontrolled immune response and cytokine storm. The acute uncontrolled innate immune response, which leads to overexpression of proinflammatory cytokines may be one of the causes for swift death in mammals infected with HPAI. Thus, one can speculate deregulation of these cytokines in chicken DCs may lead to multiple organ failure, as frequently seen in mammals.

Mx is a well-known antiviral protein, which can be induced by type 1 interferons (15). However, susceptibility to the inhibitory effects of Mx may vary by strain and host (14, 20, 54, 55). In this study, higher type 1 interferon expression levels were observed along with elevated expression of the Mx gene. However, despite the presence of elevated type 1 interferon and Mx expression levels, viral replication in DCs were not significantly inhibited. Rapid cell death and activation of caspase-dependent apoptosis did not appear to hinder the output of viral load. To date, the full complement of genes and their exact roles which contribute to antiviral properties are not well defined in chickens. However, one might speculate that the PRR dependent immune response may play a crucial role in mounting an antiviral defense, given the role of the TLR-7 and RIG-1 receptor signaling. For instance, it was shown that the presence of RIG-1 in cells stimulates expression of several key genes involved in innate immune responses that are crucial against viral infections, such as influenza (56).

Overall, we were able to demonstrate that AIV can infect and replicate in chicken DCs regardless of pathogenicity. HPAI subtypes trigger a significantly higher expression of various immune factors compared to LPAI subtypes, suggesting a dysregulation of the immune system. The increase in DC activation following infection may be indicative of the dysregulated immune responses typically seen with high pathogenic avian influenza infections.

## Data Availability

The raw data supporting the conclusions of this article will be made available by the authors, without undue reservation.
